# Correlation of oncoprotein 18/stathmin expression in human breast cancer with established prognostic factors

**DOI:** 10.1054/bjoc.2000.1264

**Published:** 2000-07-03

**Authors:** G Brattsand

**Affiliations:** Department of Clinical Chemistry, Umeå University Hospital, Umeå, S-901 85, Sweden

**Keywords:** oncoprotein 18, stathmin, breast cancer, oestrogen receptor, Western blot

## Abstract

Oncoprotein 18/stathmin (Op18) is a conserved cytosolic phosphoprotein that regulates microtubule dynamics. The microtubule destabilizing activity is regulated by phosphorylation, mediated by both growth factor stimulated- and cell-cycle regulating kinases. The protein is highly expressed in a variety of human malignancies. In human breast carcinoma, Op18 has previously been shown to be up-regulated in a subset of the tumours, however, no correlation with clinicopathologic characteristics has been reported so far. In the present study we have examined Op18 protein expression by quantitative Western blot analysis in a panel of 151 semi-consecutive breast carcinoma samples. Op18 levels were negatively correlated with oestrogen receptor (OR) expression and positively correlated with a high fraction of aneuploid cells, proliferation measured by proliferating cell nuclear antigen (PCNA) expression, tumour size and histopathologic grade. Taken together, and in contrast to what has been previously reported, the present study shows that high Op18 expression correlates with general predictive factors and is not restricted to a specific sub-group of breast carcinoma. © 2000 Cancer Research Campaign


*British Journal of Cancer* (2000) **83**(3), 311–318
© 2000 Cancer Research Campaign
doi: 10.1054/ bjoc.2000.1264, available online at http://www.idealibrary.com on IDEAL®

### Correlation of oncoprotein 18/stathmin expression in human breast cancer with established prognostic factors

**G Brattsand**

Department of Clinical Chemistry, Umeå University Hospital, S-901 85 Umeå, Sweden

**Summary** Oncoprotein 18/stathmin (Op18) is a conserved cytosolic phosphoprotein that regulates microtubule dynamics. The microtubule destabilizing activity is regulated by phosphorylation, mediated by both growth factor stimulated- and cell-cycle regulating kinases. The protein is highly expressed in a variety of human malignancies. In human breast carcinoma, Op18 has previously been shown to be up-regulated in a subset of the tumours, however, no correlation with clinicopathologic characteristics has been reported so far. In the present study we have examined Op18 protein expression by quantitative Western blot analysis in a panel of 151 semi-consecutive breast carcinoma samples. Op18 levels were negatively correlated with oestrogen receptor (OR) expression and positively correlated with a high fraction of aneuploid cells, proliferation measured by proliferating cell nuclear antigen (PCNA) expression, tumour size and histopathologic grade. Taken together, and in contrast to what has been previously reported, the present study shows that high Op18 expression correlates with general predictive factors and is not restricted to a specific sub-group of breast carcinoma. © 2000 Cancer Research Campaign

**Keywords**: oncoprotein 18; stathmin; breast cancer; oestrogen receptor; Western blot

Op18 is a 19 kDa cytosolic phosphoprotein that has been studied independently in various cellular systems under different names (e.g. p19, 19K, p18, prosolin and metablastin) (Doye et al, 1989; Schubart et al, 1989; Zhu et al, 1989; Cooper et al, 1990; Gullberg et al, 1990). The protein is conserved in vertebrates and expressed in most tissues (Sobel, 1991). High expression of Op18 has been observed in a number of human malignancies, for example acute leukaemias, lymphomas, neuroblastomas and prostatic adenocarcinomas (Hanash et al, 1988; Hailat et al, 1990; Ghosh et al, 1993; Roos et al, 1993, Luo et al, 1994; Friedrich et al, 1995). The significance of tumour-specific up-regulation of Op18 is indicated by experiments employing antisense inhibition of Op18 expression, which shows that high levels of the protein may be necessary to maintain the transformed phenotype in leukaemic cells (Jeha et al, 1996). In a recent report, Op18 was found to be overexpressed in a subset of human breast carcinomas (Bièche et al, 1998). In this study, the authors evaluated Op18 mainly at the mRNA level, but a reasonable agreement with protein expression level was reported, as measured by Western blot and immunohistochemistry. However, no significant correlations were observed between Op18 expression and established clinicopathologic parameters (Bièche et al, 1998).

Op18 has been shown to regulate microtubule dynamics both in vitro and in vivo (Belmont and Mitchison, 1996; Marklund et al, 1996). Unphosphorylated Op18 destabilizes microtubules both in vivo and in vitro and binds to soluble αβ-tubulin dimers, while phosphorylation switches off both of these activities. No interaction has so far been observed between Op18 and microtubules. The mechanism by which Op18 mediates destabilization of microtubules is currently controversial, but two distinct models have been proposed involving either sequestering of tubulin dimers or by specific catastrophe promotion, i.e. a switch from growing to shrinking microtubules (reviewed by Cassimeris, 1999; McNally, 1999).

Op18 is phosphorylated on four serine residues (Ser16, Ser25, Ser38 and Ser63) in intact cells by a wide range of extracellular effectors and during mitosis (Labdon et al, 1992; Beretta et al, 1993; Leighton et al, 1993; Marklund et al, 1993*a*; 1993*b*; 1994; Brattsand et al, 1994; Luo et al, 1994). Ser 16 and Ser63 are phosphorylated by protein kinase A, Ser16 by the $Ca^{2+}$/calmodulin-dependent kinase IV/Gr and Ser25 by members of the mitogen-activated protein kinase family (MAP/ERK) while Ser25 and Ser38 are targets for phosphorylation by cyclin-dependent kinases (reviewed by Lawler, 1998). Progression through mitosis and formation of the mitotic spindle requires multisite phosphorylation on all four serine residues (Marklund et al, 1996; Larsson et al, 1997). Op18 is implicated in microtubule regulation in response to signal transduction events during interphase of the cell cycle (Melander Gradin et al, 1997; 1998). Taken together, this indicates a central role of Op18 in microtubule regulation.

The success of microtubule-directed chemotherapeutics such as paclitaxel in cancer treatment and the abundant expression of Op18 in breast cancer, warrants further analysis of the microtubule-regulating protein Op18. The present study was initiated to evaluate Op18 expression in relation to other clinicopathologic characteristics in a larger panel of breast tumours. The results show that Op18 expression correlates with several established prognostic factors and that up-regulated expression is not restricted to any specific subgroup of breast carcinoma.

*Received 16 December 1999*
*Revised 30 March 2000*
*Accepted 10 April 2000*

*Correspondence to:* G Brattsand

### MATERIALS AND METHODS

### Patient data

The study included 151 women (median age 60 years; range 33–89). From all consecutive samples sent for OR analysis during the first 7 months of 1996 in the northern health care region of Sweden (197 totally), samples with a DNA content exceeding 62 μg were included in the study. Clinicopathologic characteristics of the patients (Table 1) were determined in clinical routine. Histopathologic grade was based on the recommendations by Elston and Ellis (1991). The number of patients for whom data were available varied among the different prognostic factors studied. The study has been approved in the ethical committee, Medical faculty, Umeå University.

### Tumour tissue preparation

During primary surgery and after pathologic examination, representative tumour tissue was cut out and sent frozen for ER analyses. Tumour samples were stored frozen in liquid nitrogen until analysed. Frozen tumour tissue was homogenized in a microdish membrator (Braun, Melsungen, Germany) and suspended in ice-cold buffer (10 mM Tris pH 7.4; 1.5 mM EDTA; 10 mM $Na_2MoO_4$; 1 mM monothioglycerol). Supernatants (centrifugation 20 000 × ***g***, 4°C, 10 min) were used for ER analyses in clinical routine. The pelleted fractions were analysed for DNA according to Burton (1968). The remaining portion of the supernatant, not consumed in the receptor analyses, was frozen at –70°C and the Western blot analyses were performed on this archive material. A volume of supernatant corresponding 6.25–16.25 μg of DNA was precipitated in 66% acetone overnight, –20°C. After centrifugation (16 000 × ***g*** for 15 min, 4°C), the protein pellet was dried under vacuum and resuspended in 25–65 μl of loading buffer (135 mM Tris pH 6.8, 2.5% SDS, 10% glycerol, 10% beta-2-mercaptoethanole and bromphenol blue) and heated for 2 min at 95°C. 20 μl of sample, corresponding to 5 μg of DNA, was loaded onto a gel.

### Protein standards and control samples

Purified recombinant Op18 with a protein concentration measured by amino acid analysis was serial diluted in six steps in loading buffer to generate a standard curve (range 1.5–49 ng per lane). 5 ng and 25 ng of Op18 per lane were used as control samples on all filters containing patient samples. Triton X-100 lysates of the cell line K562 was used as a standard curve, serially diluted in five steps from 50 μg of total protein per lane, for arbitrary quantification of PCNA.

### SDS-PAGE and Western blot

20 μl of patient samples, protein standards or control samples were loaded on 10–20% gradient SDS-PAGE gels and run on BioRad Protean II xi for approximately 400 mAh until blue front had passed through the gels (run time 5–16 h, running buffer: 2.5 mM Tris; 0.2 mM glycine; 0.1% SDS). Electroelution in a Trans-blot cell (plate electrodes, BioRad) to pre-wetted nitrocellulose filters (Hybond ECL, Amersham) in buffer (20 mM Tris; 150 mM glycine; 20% methanol; 0.01% SDS) for 4 h at 200 mA, 4°C. Filters were washed 3 × 5 min in phosphate buffered saline (PBS), stained for 15 s (50% methanol; 0.05% Coomassie blue) to visualize protein pattern and molecular weight standards (LMW calibration kit, Pharmacia), destained for 2 min (50% methanol; 10% acetic acid) and washed for 2 × 5 min in PBS. Filters were dried overnight at room temperature. Strips corresponding to Op18 (19 kD), triose phosphate isomerase (28 kD) and PCNA (34 kD) were cut from filters and blocked in blocking buffer (5% non-fat dried milk; 0.03% antifoam in PBS) for 5 h at room temperature. For detection of Op18, strips were incubated with anti Op18 rabbit antiserum (1/100) in blocking buffer. PCNA was detected by the monoclonal antibody PC10 (DACO) 2 μg $ml^{-1}$ in 15% foetal calf serum (FCS); 150 mM NaCl; 2.5 mM EDTA; 50 mM Tris pH 7.5; 0.02% $NaN_3$. Antibody incubations were carried out overnight at 4°C on a rocking table in sealed plastic bags with 3.5 ml antibody solution per strip. Filters were then washed for 2 min in TBS pH 7.5 (20 mM Tris pH 7.5; 137 mM NaCl), 3 × 2 min in TBS-T pH 7.5 (0.1% Tween-20 in TBS) and 2 × 2 min in TBS pH 7.5. After primary antibody incubations and washing, strips were incubated with $^{125}$I-protein A in 20% FCS; 150 mM NaCl; 2.5 mM EDTA; 50 mM Tris pH 8.8; 0.02% $NaN_3$ for 30 min at room temperature. Filters were then washed 2 × 2 min in TBS pH 8.0, 5 × 2 min in TBS-T pH 8.0 and 3 × 2 min in TBS pH 8.0. Filters were dried at room temperature for 3 h, exposed on BioRad SI screens and analysed on BioRad phosphoimager GS-525 Molecular Imager System. Exposure time was for 2–20 h (depending on antibody) to allow use of the dynamic range of the screen without saturation. Immunoreactive bands on the digital image were marked with rectangles of the same size. Counts within the rectangle (volume counts × $mm^2$) were used for calculation of concentrations based on the standard curve within

**Table 1** Clinicopathologic characteristics of the patients

| Variable | No of patients |
|---|---|
| Total patients (*n*) | 151 |
| Histologic type | |
| Ductal invasive | 72 |
| Lobular invasive | 5 |
| Others | 4 |
| Not analysed | 70 |
| Tumour size | |
| T1 (≤ 20 mm) | 42 |
| T2 (> 20 mm) | 31 |
| Not analysed | 78 |
| Histopathologic grade | |
| I | 12 |
| II | 35 |
| III | 31 |
| Not analysed | 73 |
| Node status | |
| Node negative | 50 |
| Node positive | 20 |
| Not analysed | 81 |
| OR status | |
| OR + (≥ 0.1 fmol $\mu g^{-1}$ DNA) | 106 |
| OR – (< 0.1 fmol $\mu g^{-1}$ DNA) | 45 |
| Ploidy | |
| Normoploid | 14 |
| Aneuploid | 52 |
| Not analysed | 85 |
| S-phase fraction | |
| ≤ 8% | 44 |
| > 8% | 14 |
| Not analysed | 93 |

*© 2000 Cancer Research Campaign*

each series. Op18 concentrations were recalculated as ng μg$^{-1}$ DNA.

### OR and progesterone receptor (PgR) analysis

OR and PgR content was determined by an enzyme immunoassay (Abbott Lab, IL, USA). Receptor concentration was expressed as fmol μg$^{-1}$ DNA. Tumours with a value < 0.1 fmol μg$^{-1}$ DNA were considered receptor-negative and those with a value ≥ 0.1 as receptor-positive.

### S-phase fraction, DNA ploidy and fraction of aneuploid cells

Cell suspensions were prepared from frozen tissue and used for DNA histogram analyses. DNA staining was done according to Vindelov et al (1983) and analyses were performed on a FACScan instrument (Becton Dickinson, CA, USA). S-phase fraction and DNA index was calculated by Cellfit software (Becton Dickinson) in RFIT model, when possible, or calculated manually. DNA histograms were classified as diploid near diploid when only one $G_0/G_1$ peak was detected at DNA index 1.0, and as aneuploid when additional peaks were identified. The fraction of aneuploid cells, in the samples classified as aneuploid, was estimated based on comparison of the aneuploid $G_0/G_1$ peak and the diploid $G_0/G_1$ peak.

### Cell lines

Breast cancer cell lines obtained from American Type Culture Collection (ATCC) were grown in RPMI 1640 medium supplemented with 10% FCS and antibiotics. Cells were harvested with trypsin, frozen and processed as the tumour samples.

### Statistical analyses

Association between Op18 levels and established predictive factors was evaluated by the Mann–Whitney rank test, with Op18 as continuous variable. Cut-offs for the group dividing variables were according to guidelines of the North Swedish Breast Cancer Group. Comparison of PCNA level, the fraction of aneuploid cells, DNA index and Op18 were evaluated by the Mann–Whitney rank test with Op18 as group-dividing variable with a cut-off value of 0.6 ng Op18 μg$^{-1}$ DNA. The level of significance for rejecting the null hypothesis of zero effect was taken to be $P = 0.05$. All calculations were performed in SPSS version 9.0 (SPSS, IL, USA).

### RESULTS

### Op18 expression in tumour samples

151 tumour specimens from breast cancer patients were analysed for Op18 expression. This panel of specimens contained samples from patients with primary, unilateral stage I–III disease and also in a few cases samples came from patients with actual or previously diagnosed cancer in the same or opposite breast and/or stage IV disease. Clinicopathologic characteristics of the patients are outlined in Table 1. Determination of Op18 mass concentrations, expressed as ng μg$^{-1}$ of DNA, were performed by Western blot and quantified by phosphoimager as described in the Materials and Methods section. Representative exposures of Western blots are

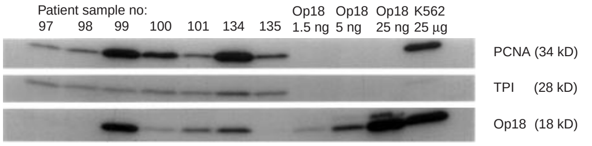

**Figure 1** Representative Western blots of protein extracts from seven breast cancer samples, three standard levels of recombinant Op18 protein and the cell line K562 using antibodies to PCNA, TPI and Op18 (an extra band with slightly higher molecular weight is seen in the Op18 blot in the Op18 control samples, this is due to inclusion of a tagged variant of recombinant Op18 and is without significance for the current study)

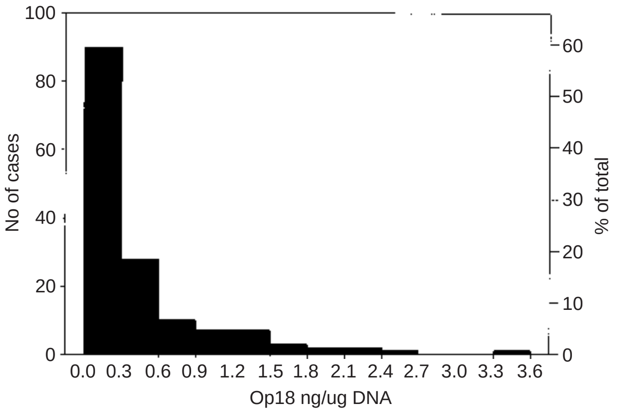

**Figure 2** Frequency distribution histogram of the Op18 level in 151 breast cancer samples. The left vertical axis represents number of cases in each concentration interval and the right vertical axis represents percent of total number of cases. The limit of quantitative measurement was defined as 0.3 ng of Op18 per μg of DNA and the cut-off level used in statistical analysis was 0.6 ng of Op18 per μg of DNA

shown in Figure 1. The Op18 antiserum used detected a single band only at 19 kD in the patient material as described earlier (Brattsand et al, 1993). An extra band with slightly higher molecular weight is seen in the Op18 control samples in Figure 1. This is because an epitope-tagged recombinant Op18 protein with eight extra amino acids was mixed with native Op18 in the samples used as control. This band is without significance for the current study and is not included in the quantitative measurements.

The Op18 expression varied considerably among the tumours as seen in Figure 1 and the frequency distribution histogram in Figure 2. Some tumours exhibited at least 10–20 times higher Op18 concentration than others. Western blot of the glycolytic enzyme triose-phosphate isomerase (TPI) was used as an internal control and showed only minor variations (Figure 1). Only a faint TPI band is seen in the K562 lysate in Figure 1 since the total loaded protein amount is less than in the patient samples. It is evident from Figure 2 that the frequency distribution of Op18 is considerably asymmetric. The limit of quantitative measurement was defined as 0.3 ng μg$^{-1}$ DNA since this corresponds to an Op18 standard concentration giving rise to a signal higher than the mean background signal plus three times the standard deviation (SD) of the background signal. However, the concentrations have been extrapolated at even lower values. The Western blot method used to quantify Op18 protein levels was validated and showed a total coefficient of variation (CV) of around 30% for Op18 standards and patient samples (data not shown). This means that two quantitative measurements are separate with 95% confidence when they

© 2000 Cancer Research Campaign

**Table 2** Rank test with Op18 as continuous variable

| Variable | $n^a$ | Mean rank Op18[b] | *P*-value[c] Asymp.sig. (2-tailed) |
|---|---|---|---|
| OR expression | | | |
| OR + (≥ 0.1 fmol µg$^{-1}$ DNA) | 106 | 68.4 | 0.001 |
| OR – (< 0.1 fmol µg$^{-1}$ DNA) | 45 | 94.0 | |
| PgR expression | | | |
| PgR + (≥ 0.1 fmol µg$^{-1}$ DNA) | 96 | 67.4 | 0.001 |
| PgR – (< 0.1 fmol µg$^{-1}$ DNA) | 55 | 91.0 | |
| Size | | | |
| T1 (≤ 20 mm) | 42 | 32.0 | 0.020 |
| T2 (> 20 mm) | 31 | 43.7 | |
| Histopathologic grade | | | |
| I + II | 47 | 35.0 | 0.030 |
| III | 31 | 46.3 | |
| S-phase fraction | | | |
| ≤ 8% | 44 | 27.4 | 0.092 |
| > 8% | 14 | 36.1 | |
| Node status | | | |
| Negative | 50 | 35.1 | 0.800 |
| Positive | 20 | 36.5 | |
| Ploidy | | | |
| Normoploid | 14 | 34.0 | 0.912 |
| Aneuploid | 52 | 33.4 | |

[a]Number of patients with accessible data for the group in the variable, [b]mean rank of Op18 expression for the group in the variable, [c]Mann–Whitney U test.

**Table 3** Rank test with Op18 as group dividing variable

| Variable | $n^a$ | Mean rank of variable[b] | *P*-value[c] Asymp.sig. (2-tailed) |
|---|---|---|---|
| PCNA | | | |
| Op18 ≤ 0.6 ng µg$^{-1}$ DNA | 118 | 67.6 | <0.001 |
| Op18 > 0.6 ng µg$^{-1}$ DNA | 33 | 106.1 | |
| Fraction of aneuploid cells | | | |
| Op18 ≤ 0.6 ng µg$^{-1}$ DNA | 40 | 22.7 | 0.003 |
| Op18 > 0.6 ng µg$^{-1}$ DNA | 11 | 37.9 | |
| DNA index | | | |
| Op18 ≤ 0.6 ng µg$^{-1}$ DNA | 39 | 26.1 | 0.956 |
| Op18 > 0.6 ng µg$^{-1}$ DNA | 12 | 25.8 | |

[a]Number of patients with accessible data for the two groups, [b]mean rank of the variable PCNA expression, fraction of aneuploid cells (%) and DNA index, respectively for the two groups, [c]Mann–Whitney U test.

differ by a factor of around two (Sadler et al, 1992). As seen in Figure 2, the median Op18 expression was below 0.3 ng µg$^{-1}$ DNA, i.e. below the limit of quantitative measurement. By using a cut-off that is twice the limit of quantitative measurement, i.e. 0.6 ng µg$^{-1}$ DNA, it is possible to separate a group of tumours that show higher and more varied Op18 expression than the rest. This group represents 22% of the samples and is defined as a group of tumours with up-regulated Op18 expression. This cut-off was used in the subsequent statistical analysis.

### Relationship between Op18 and other parameters

Associations between Op18 levels and other accessible biological and clinical parameters are shown in Tables 2 and 3 for the 151 patients. A significant relationship exists between loss of OR expression and Op18 level ($P$ = 0.001). This relationship was also significant in Person's $\chi^2$ test when a cut-off Op18 of 0.6 ng µg$^{-1}$ DNA was used ($P$ = 0.002). The inverse relationship between Op18 and OR expression is illustrated in Figure 3. A similar negative correlation was observed between Op18 and progesterone-receptor expression (Table 2). A significant positive correlation was seen between tumour size and Op18 levels, as well as between histopathologic grade and Op18 levels (Table 2). Expression of Op18 was significantly correlated to PCNA expression, arbitrarily quantified by Western blot as described in the Materials and Methods section (Table 3). The level of Op18 was also compared with the fraction of aneuploid cells in the tumour samples harbouring aneuploid populations (Table 3 and Figure 4). It is evident that the tumour samples showing the highest Op18 levels also harbour a high fraction of aneuploid cells. No correlations were seen between Op18 levels and node status, ploidy status, DNA index (Table 2 and 3) or age (not shown).

 © 2000 Cancer Research Campaign

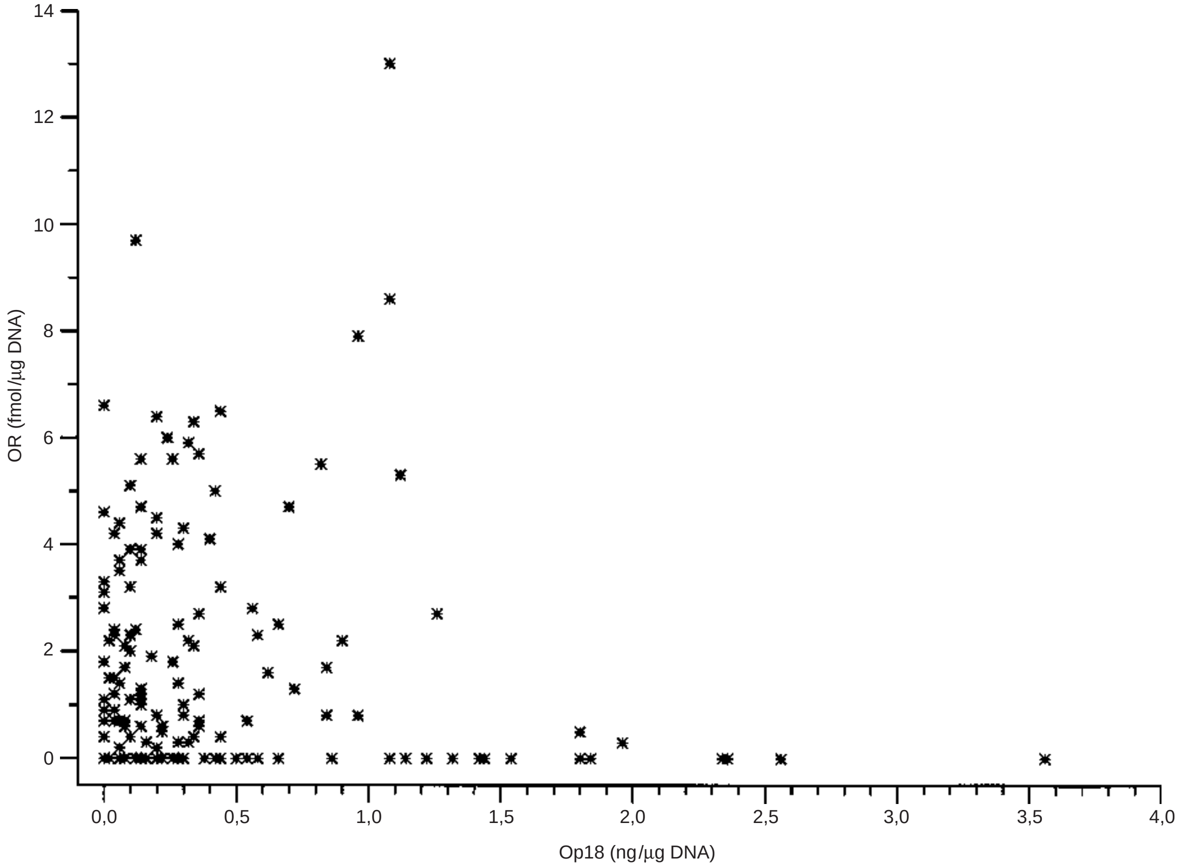

**Figure 3** Op18 concentrations vs OR concentrations in 151 breast cancer samples

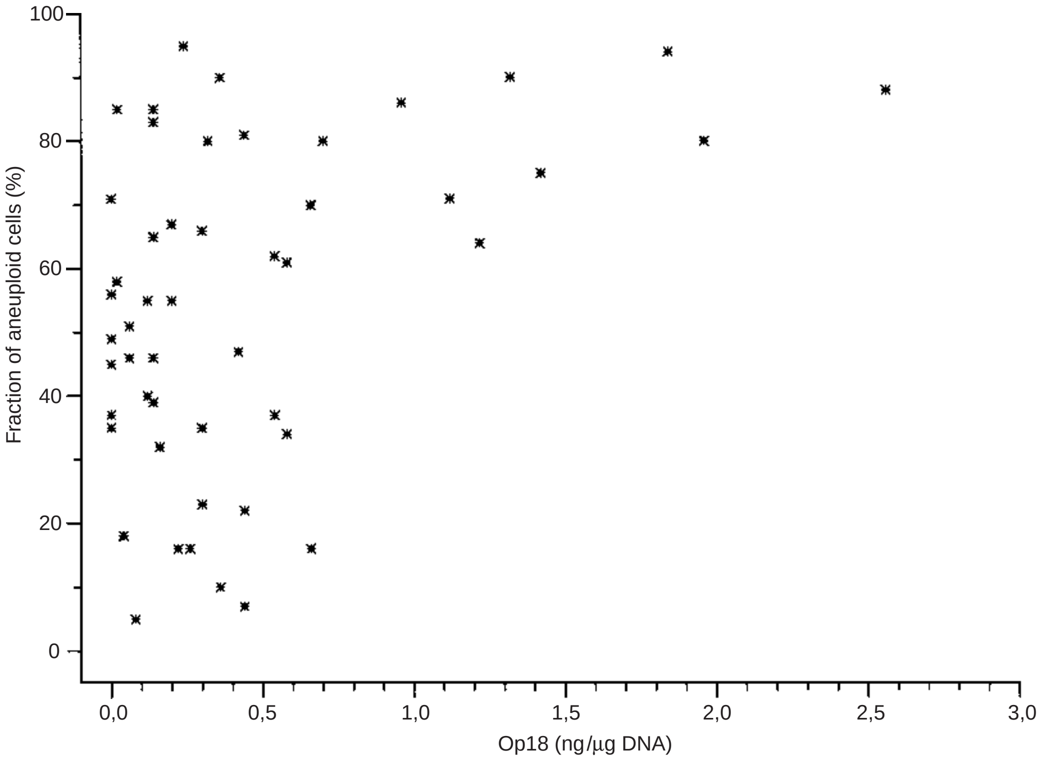

**Figure 4** Op18 concentrations vs the fraction of aneuploid cells (expressed as %) in 51 breast cancer samples harbouring aneuploid populations

*© 2000 Cancer Research Campaign* 

**Table 4** Op18 expression in breast cancer cell lines

| Cell line | OR (pmol μg$^{-1}$ DNA) | Op18 (ng μg$^{-1}$ DNA) |
|---|---|---|
| MDA-MB 468 | 0.0 | 7.6 |
| MDA-MB 231 | 0.0 | 4.7 |
| CAMA-1 | 0.2 | 3.8 |
| T-47D1 | 0.3 | 3.2 |
| MCF-7 | 0.2 | 1.2 |

### Op18 expression in breast cancer cell lines

To determine if up-regulated Op18 expression is a constitutive feature of OR-negative breast cancer cells, five cell lines of breast cancer origin were investigated for Op18 expression. The cell lines MDA-MB 468 and MDA-MB 231 (Cailleau et al, 1978) were confirmed to be OR-negative and the cell lines MCF-7, T-47D1 and CAMA-1 (Soule et al, 1973; Fogh et al, 1977; Keydar et al, 1979) were confirmed to be OR-positive (Table 4). The mean of Op18's quadruple determination in the five cell lines are shown in Table 4. The OR-negative cell lines showed the highest Op18 expression, indicating that up-regulated Op18 expression is present even after long-term in vitro culture of OR-negative breast cancer cells. It is apparent from Figure 2 and Table 4 that the tumour samples showing the highest Op18 expression have similar levels to the ones seen in cell lines. Calculation of Op18 expression per total protein amount did not alter the interrelationship between the cell lines (data not shown).

### DISCUSSION

The quantitative Western blot data in this study are based on measurements of DNA content in the samples. Protein content has been measured in 127 of the 151 patient samples and correlates with DNA content (correlation coefficient (*r*) of 0.66). In average, based on linear regression, 1 μg of DNA corresponds to 10 μg of total protein (data not shown). Quantification per DNA may be more specific than per protein, due to the influence of extracellular and blood proteins in the biopsies. Samples with a high DNA index (i.e. polyploid) did not show any consistent bias towards lower relative protein amount (data not shown). Recalculation of the quantitative measurements per protein did not induce any gross alterations in the observed correlation with clinicopathologic parameters.

Control samples from normal breast tissue have not been available for this study. Aneuploid cells in a tumour sample can, for good reasons, be regarded as malignant. The observation that a high level of Op18 is seen in samples with a high fraction of aneuploid cells (Figure 4) shows that Op18 is mainly expressed by the malignant cells in the samples and the level of Op18 measured by Western blot reflects both the expression of individual tumour cells and the fraction of tumour cells in the samples. In an earlier study by Bièche et al (1998), control samples showed low Op18 expression on the mRNA level and infiltrating lymphocytes were shown to express weak or no Op18 immunoreactivity upon immunohistochemical stainings. Taken together, these lines of evidence show that a high Op18 level reflects specific expression by tumour cells and that at least some breast carcinoma cells have an up-regulated Op18 expression. We can define a group of breast carcinomas representing 22% of the cases, that with statistical confidence show up-regulated Op18 expression. In the study by Bièche et al (1998), 15 of 50 breast carcinoma samples were defined to overexpress Op18 on the mRNA level. In that study the Op18 protein concentration range (seven samples) was broader than we found, however comparison is difficult since no mass unit was used in the earlier study.

High Op18 levels correlate with loss of OR (Figure 3). In agreement with the finding in patient material, the OR-negative cell lines MDA-MB 468 and MDA-MB 231 show higher Op18 levels than the OR-positive cell lines CAMA-1, T47-D1 and MCF 7 (Table 4). Several other factors are also known to correlate with loss of OR, for example proliferation (Fechter et al, 1988; Sigurdsson et al, 1990; Meyer and Province, 1994), overexpression of cycline E (Nielsen et al, 1996), and mutations in the *p53* gene (Caleffi et al, 1994).

To evaluate the proliferative status in the tumour samples, PCNA was quantified by Western blot. Levels of Op18 correlate with PCNA levels (Table 3) which indicates an association with proliferation in breast carcinoma. The fraction of cells in S-phase shows a weak correlation with Op18 levels, although this correlation was not statistically significant (Table 2). Whether Op18 expression in general is linked to proliferation seems to depend on the cell/tissue/tumour system studied (Brattsand et al, 1993; Koppel et al, 1993; Roos et al, 1993; Nylander et al, 1995; Balogh et al, 1996).

It has been speculated that defective mitotic spindle checkpoints and aberrant regulation of centrosomes are involved in chromosomal genetic instability (reviewed for example by Lengauer et al, 1998, Zimmerman et al, 1999). An obvious question is of course whether Op18 could be involved in such processes considering its regulatory effects on microtubule dynamics. However, Op18 levels do not show any correlation with the ploidy status of the tumours (Table 2), and this argues against such an involvement. Op18 levels do not show any correlation with lymph node engagement (Table 2) and this argues against Op18 being involved in the metastatic process.

The reason for the up-regulated expression of Op18 in tumour cells has remained elusive. However, recent findings suggest the *Op18* gene to be transcriptionally repressed by a p53/histone deacetylase complex (Ahn et al, 1999; Murphy et al, 1999). The up-regulated Op18 expression in tumour cells may thus, at least in part, reflect a defective p53/histone deacetylase signalling function. This is in line with the observation that Op18 expression correlates with loss of OR expression, which in turn correlates with mutations in the p53 gene (Caleffi et al, 1994).

To conclude, in contrast to what has previously been reported, the present study shows that high Op18 expression correlates well with general prognostic factors and is not restricted to a specific subgroup of breast carcinomas. Further investigations on the usefulness of Op18 as a prognostic marker for disease-free survival and overall survival in breast carcinomas are in process. The association between Op18 expression and malignancy in breast carcinoma and other tumour types makes the protein of further interest to study in the context of tumourogenesis involving cytoskeletal alterations, p53 signalling, apoptosis, and response to chemotherapy, considering its role in microtubule regulation. For example, Op18 partially antagonizes the microtubule stabilizing effect of paclitaxel in the cell line K562 (Marklund et al, 1996), and therefore it cannot be excluded that Op18 may be a resistance factor for microtubule-directed chemotherapy.

Interestingly, the expression of microtubule-associated protein 4 (MAP4), a protein also transrepressed on transcriptional level by p53(Murphy et al, 1999), affects the sensitivity to antimicrotubule

*© 2000 Cancer Research Campaign*

drugs (Zhang et al, 1998; 1999). It has been speculated that microtubule regulation is an important downstream event during p53-dependent induction of apoptosis (Ahn et al, 1999; Murphy et al, 1999). Such a pathway, influenced by p53-mediated repression of both Op18 and MAP4, may be of importance for treatment of cancer patients with microtubule-directed chemotherapy.

### ACKNOWLEDGEMENTS

Anita Huurala, Karin Karvonen and Bodil Bäckström are acknowledged for skilled technical assistance. Jari Norvanto is acknowledged for fruitful collaboration, Björn Tavelin for excellent statistical support and Brigette Bilbe for linguistic corrections. Martin Gullberg and Kjell Grankvist are acknowledged for fruitful discussions and for critical reading of the manuscript. This work was supported by grants from the research foundation of the department of oncology, Umeå University, Margareta Danneborgs foundation, Medical faculty, Umeå University and the Swedish Medical Research Council (K1999–32P-13116–01A).

### Note added in proof

After the submission of this report, a study concerning Op18 in breast cancer has been published (Curmi et al, *Br J Cancer* **82**: 142–150). In this study Op18 is measured on mRNA level by competitive RT-PCR and correlates to loss of steroid receptors and histopathologic grade, which agrees with the conclusions of the present study.

### REFERENCES

Ahn J, Murphy M, Kratowicz S, Wang A, Levine AJ and George DL (1999) Down regulation of the stathmin/Op18 and FKBP25 genes following p53 induction. *Oncogene* **18**: 5954–5958

Balogh A, Mege RM and Sobel A (1996) Cell density dependent expression of stathmin in C2 myoblasts in culture. *Exp Cell Res* **224**: 8–15

Belmont LD and Mitchison TJ (1996) Identification of a protein that interacts with tubulin dimers and increases the catastrophe rate of microtubules. *Cell* **84**: 623–631

Beretta L, Dobransky T and Sobel A (1993) Multiple phosphorylation of stathmin. Identification of four sites phosphorylated in intact cells and in vitro by cyclic AMP-dependent protein kinase and p34cdc2. *J Biol Chem* **268**: 20076–20084

Bièche I, Lachkar S, Becette V, Cifuentes-Diaz C, Sobel A, Lidereau R and Curmi PA (1998) Overexpression of the stathmin gene in a subset of human breast cancer. *Br J Cancer* **78**: 701–709

Brattsand G, Roos G, Marklund U, Ueda H, Landberg G, Nånberg E, sideras P and Gullberg M (1993) Quantitative analysis of the expression and regulation of an activation-regulated phosphoprotein (oncoprotein 18) in normal and neoplastic cells. *Leukemia* **7**: 569–579

Brattsand G, Marklund U, Nylander K, Roos G and Gullberg M (1994) Cell-cycle-regulated phosphorylation of oncoprotein 18 on Ser16, Ser25 and Ser 38. *Eur J Biochem* **220**: 359–368

Burton K (1968) Determination of DNA concentrations with diphenylamine. In: *Methods in enzymology*, Vol. 12, part B, Grossman L, Moldave K (eds), pp. 163–166. Academic Press: New York

Cailleau R, Olivé M and Cruciger QVJ (1978) Long-term human breast carcinoma cell lines of metastatic origin: Preliminary characterization. *In Vitro* **14**: 911–915

Caleffi M, Teague MW, Jensen RA, Vnencak-Jones CL, Dupont WD and Parl FF (1994) P53 gene mutations and steroid receptor status in breast cancer. *Cancer* **73**: 2147–2156

Cassimeris L (1999) Accessory protein regulation of microtubule dynamics throughout the cell cycle. *Curr Opin Cell Biol* **11**: 134–141

Cooper HL, Fulder R, McDuffie E and Braverman R (1990) A specific defect of prosolin phosphorylation in T cell leukemic lymphoblasts is associated with impaired down-regulation of DNA synthesis. *J Immunol* **145**: 1205–1213

Doye V, Soubrier F, Bauw G, Boutterin MC, Beretta L, Koppel J, Vandekerckhove J and Sobel A (1989) A single cDNA encodes two isoforms of stathmin, a developmentally regulated neuron-enriched phosphoprotein. *J Biol Chem* **264**: 12134–12137

Elston CW and Ellis IO (1991) Pathological prognostic factors in breast cancer, I. The value of histological grade in breast cancer: experience from a large study with long-term follow-up. *Histopathology* **19**: 403–410

Fechter GE, Mueller A, Kaufmann M, Haag D, Born IA, Abel U, Klinga K, Kubli F and Goerttler K (1988) Correlation of DNA flow cytometric results and other prognostic factors in primary breast cancer. *Int J Cancer* **41**: 823–828

Fogh J, Wright WC and Loveless JD (1977) Absence of HeLa cell contamination in 169 cell lines derived from human tumours. *J Natl Cancer Inst* **58**: 209–214

Friedrich B, Grönberg H, Landström M, Bergh A and Gullberg M (1995) Differentiation-stage specific expression of oncoprotein 18 in human and rat prostatic adenocarcinoma. *Prostate* **27**: 102–109

Ghosh PK, Anderson J, Cohen N, Takeshita K, Atweh GF and Lebowitz P (1993) Expression of the leukemia-associated gene, p18, in normal and malignant tissues; inactivation of expression in a patient with cleaved B-cell lymphoma/leukemia. *Oncogene* **8**: 2869–2872

Gullberg M, Noreus K, Brattsand G, Friedrich B and Shingler V (1990) Purification and characterization of a 19-kilodalton intracellular protein. An activation-regulated putative protein kinase C substrate of T lymphocytes. *J Biol Chem* **265**: 17499–17505

Hailat N, Strahler JR, Melhem RF, Zhu XX, Brodeur G, Seeger RC, Reynolds CP and Hanash SM (1990) N-myc gene amplification in neuroblastoma isassociated with altered phosphorylation of a proliferation related polypeptide (Op18). *Oncogene* **5**: 1615–1618

Hanash SM, Strahler JR, Kuick R, Chu EH and Nichols D (1988) Identification of a polypeptide associated with the malignant phenotype in acute leukemia. *J Biol Chem* **263**: 12813–12815

Jeha S, Luo X, Beran M, Kantarjian H and Atweh GF (1996) Antisense RNA inhibition of phosphoprotein p18 expression abrogates the transformed phenotype of leukemic cells. *Cancer Res* **56**: 1445–1450

Keydar I, Chen L, Karby S, Weiss FR, Delarea J, Radu M, Chaitcik S and Brenner HJ (1979) Establishment and characterization of a cell line of human breast carcinoma origin. *Eur J Cancer* **15**: 659–670

Koppel J, Loyer P, Maucuert A, Rehák P, Manceau V, Guguen-Guillouzo C and Sobel A (1993) Induction of stathmin expression during liver regeneration. *FEBS Lett* **331**: 65–70

Labdon JE, Nieves E and Schubart UK (1992) Analysis of phosphoprotein p19 by liquid chromatography/mass spectrometry. Identification of two proline-directed serine phosphorylation sites and a blocked amino terminus. *J Biol Chem* **267**: 3506–3513

Larsson N, Marklund U, Melander Gradin H, Brattsand, G and Gullberg M (1997) Control of microtubule dynamics by oncoprotein 18: Dissection of the regulatory role of multisite phosphorylation during mitosis. *Mol Cell Biol* **17**: 5530–5539

Lawler S (1998) If you need a shrink try stathmin/Op18. *Curr Biol* **8**: 212–214

Leighton IA, Curmi P, Campbell DG, Cohen P and Sobel A (1993) The phosphorylation of stathmin by MAP kinase. *Mol Cell Biochem* **127–128**: 151–156

Lengauer C, Kinzler KW and Vogelstein B (1998) Genetic instabilities in human cancers. *Nature* **396**: 643–649

Luo X, Mookerjee B, Ferrari A, Mistry S and Atweh GF (1994) Regulation of phosphoprotein p18 in leukemic cells. Cell cycle regulated phosphorylation by $p34^{cdc2}$ kinase. *J Biol Chem* **269**: 10312–10318

Marklund U, Brattsand G, Osterman O, Ohlsson PI and Gullberg M (1993*a*) Multiple signal transduction pathways induce phosphorylation of serine 16, 25, and 38 of oncoprotein 18 in T lymphocytes. *J Biol Chem* **268**: 25671–25680

Marklund U, Brattsand G, Shingler V and Gullberg M (1993*b*) Serine 25 of oncoprotein 18 is a major cytosolic target for the mitogen-activated protein kinase. *J Biol Chem* **268**: 15039–15047

Marklund U, Larsson N, Brattsand G, Osterman Ö, Chatila TA and Gullberg M (1994) Serine 16 of oncoprotein 18 is a major cytosolic target for the $Ca^{2+}$/calmodulin-dependent kinase-Gr. *Eur J Biochem* **225**: 53–60

Marklund U, Larsson N, Melander Gradin H, Brattsand G and Gullberg M (1996) Oncoprotein 18 is a phosphporylation-responsive regulator of microtubule dynamics. *EMBO J* **15**: 5290–5298

McNally FJ (1999) Controlling split ends. *Curr Biol* **9**: 274–276

Melander Gradin H, Marklund U, Larsson N, Chatila TA and Gullberg M (1997) Regulation of microtubule dynamics by $Ca^{2+}$/calmodulin-dependent kinase IV/Gr-dependent phosphorylation of oncoproten 18. *Mol Cell Biol* **17**: 3459–3467

Melander Gradin H, Larsson N, Marklund H and Gullberg M (1998) Regulation of Microtubule dynamics by extracellular signals: cAMP-dependent protein kinase switches off the activity of oncoprotein 18 in intact cells. *J Cell Biol* **140**: 131–141
Meyer JS and Province MA (1994) S-phase fraction and nuclear size in long term prognosis of patients with breast cancer. *Cancer* **74**: 2287–2299
Murphy M, Ahn J, Walker KK, Hoffman WH, Evans RM, Levine AJ and George DL (1999) Transcriptional repression by wild-type p53 utilizes histone deacetylases, mediated by interaction with mSin3a. *Genes Dev* **13**: 2490–2501
Nielsen NH, Arnerlöv C, Emdin SO and Landberg G (1996) Cyclin E overexpression, a negative prognostic factor in breast cancer with strong correlation to ostrogen receptor status. *Br J Cancer* **74**: 874–880
Nylander K, Marklund U, Brattsand G, Gullberg M and Roos G (1995) Immunohistochemical detection of oncoprotein 18 (Op18) in malignant lymphomas. *Histochem J* **27**: 155–160
Roos G, Brattsand G, Landberg G, Marklund U and Gullberg M (1993) Expression of oncoprotein 18 in human leukemias and lymphomas. *Leukemia* **7**: 1538–1546
Sadler WA, Murray LM and Turner JG (1992) Minimum distinguishable difference in concentration: A clinically oriented translation of assay precision summaries. *Clin Chem* **38**: 1773–1778
Schubart UK, Banerjee MD and Eng J (1989) Homology between the cDNAs encoding phosphoprotein p19 and SCG10 reveals a novel mammalian gene family preferentially expressed in developing brain. *DNA* **8**: 389–398
Sigurdsson H, Baldetorp B, Borg Å, Dalberg M, Fernö M, Killander D and Olsson H (1990) Indicators of prognosis in nod-negative breast cancer. *N Engl J Med* **322**: 1045–1053
Sobel A (1991) Stathmin: a relay phosphoprotein for multiple signal transduction? *Trends Biochem Sci* **16**: 301–305
Soule HD, Vazquez J, Long A, Albert S and Brennan M (1973) A human cell line from a pleural effusion derived from a breast carcinoma. *J Natl Cancer Inst* **21**: 1409–1416
Vindelov LL, Christensen IJ, Nissen NI (1983) A detergen-trypsin method for the preparation of nuclei for flow cytometric DNA analysis. *Cytometry* **3**: 323–327
Zhang CC, Yang JM, White E, Murphy M, Levine A and Hait WN (1998) The role of MAP4 expression in the sensitivity to paclitaxel and resistance to vinca alkaloids in p53 mutant cells. *Oncogene* **16**: 1617–1624
Zhang CC, Yang JM, Bash-Babula J, White E, Murphy M, Levine AJ and Hait NW (1999) DNA damage increases sensitivity to vinca alkaloids and decreases sensitivity to taxanes through p53-dependent repression of microtubule-associated protein 4. *Cancer Res* **59**: 3663–3670
Zhu XX, Kozarsky K, Strahler JR, Eckerskorn C, Lottspeich F, Melhem R, Lowe J, Fox DA, Hanash SM and Atweh GF (1989) Molecular cloning of a novel human leukemia-associated gene. Evidence of conservation in animal species. *J Biol Chem* **264**: 14556–14560
Zimmerman W, Sparks CA and Doxsey SJ (1999) Amorphous no longer: the centrosome comes into focus. *Curr Opin Cell Biol* **11**: 122–128

 *© 2000 Cancer Research Campaign*
